# Task-State fMRI-Derived Whole-Brain Functional Topology-Constrained Spiking Neural Network with an Embedded Auditory Core Circuit for Speech Recognition

**DOI:** 10.3390/biomimetics11070481

**Published:** 2026-07-09

**Authors:** Lei Guo, Yaxin Yang

**Affiliations:** 1 Tianjin Key Laboratory of Bioelectromagnetic Technology and Intelligent Health, School of Health Sciences and Biomedical Engineering, Hebei University of Technology, Tianjin 300131, China; 202432903023@stu.hebut.edu.cn; 2 State Key Laboratory of Intelligent Power Distribution Equipment and System, Hebei University of Technology, Tianjin 300401, China

**Keywords:** spiking neural network, task-state fMRI, speech recognition, whole-brain functional topology, auditory core circuit

## Abstract

The topology of spiking neural networks (SNNs) plays an important role in determining their dynamic representation ability, recognition performance, and biological interpretability in speech recognition. However, most existing SNN reservoirs are constructed using random, regular, or manually designed connectivity patterns, which may not reflect the functional organization of the human brain during speech perception. In this study, we propose a task-state fMRI-constrained SNN framework for speech recognition. Human fMRI data acquired during naturalistic English audiobook listening are used offline to derive a task-state whole-brain functional topology, which serves as a biologically inspired structural prior for the recurrent connectivity of the SNN reservoir. Because the fMRI and downstream isolated-digit recognition tasks use different speech paradigms, this topology is interpreted as a general speech-listening prior rather than a digit-specific neural representation. The Schaefer-400 cortical parcellation is used to define 400 whole-brain functional nodes, all of which are retained to preserve distributed cortical interactions during speech listening. Within this topology, 7 SomMotB_Aud parcels are identified as auditory core nodes and analyzed as an embedded auditory circuit. Compared with resting-state fMRI, task-state fMRI shows enhanced functional connectivity among these auditory nodes, indicating task-related auditory-circuit activation. The resulting 400-node task-state topology is mapped onto the recurrent connectivity of the SNN reservoir. This mapping is regarded as a topology-constrained computational abstraction rather than a direct model of biological information transmission. During recognition, speech spike trains are the only external input, while fMRI data are used only for offline topology construction. Experimental comparisons with baseline SNNs show that the proposed topology improves recognition performance and biological interpretability. Resting-state topology comparison, auditory-core contribution analysis, threshold-sensitivity analysis, and statistical testing are further used to evaluate robustness. These findings suggest that speech-evoked whole-brain functional organization may provide an effective topology prior for biologically inspired speech recognition models.

## 1. Introduction

Speech recognition is a fundamental task in auditory information processing and human–computer interaction [1,2]. In recent years, deep neural networks have achieved remarkable performance in speech recognition [3]. However, most existing models rely on dense artificial architectures and large-scale training, which differ substantially from the sparse, event-driven, and biologically organized processing mechanisms of the human brain [4,5]. Spiking neural networks (SNNs), as the third generation of neural networks, represent information using discrete spike events and provide a biologically inspired computational framework for temporal signal processing [6]. Due to their event-driven dynamics and temporal coding capability, SNNs have attracted increasing attention in speech recognition tasks [7,8,9]. Recent SNN-based speech perception studies have further shown that recurrent spiking dynamics and neural oscillatory mechanisms can support speech-processing representations [10].

In SNN-based speech recognition, the reservoir topology plays an important role in determining the dynamic representation ability of the network [11]. Conventional SNN reservoirs are usually constructed using random, regular, small-world, or manually designed connectivity patterns [12]. Although these topologies can generate rich temporal dynamics, they are not directly derived from the functional organization of the human auditory system [13,14,15]. As a result, the biological interpretability of the reservoir structure remains limited, and the network topology may not be optimally matched to the neural mechanisms involved in speech perception [7,16]. Recent fMRI-constrained SNN studies have attempted to improve biological interpretability by constructing an SNN topology from human functional brain networks [17].

Human speech perception is supported by distributed brain networks [18]. Although auditory cortical regions play a central role in processing acoustic speech signals, other cortical regions may also contribute to attention, temporal integration, cross-regional coordination, and task-related information processing [19,20]. Therefore, a biologically meaningful SNN topology for speech recognition should not only emphasize auditory-related circuits but also preserve broader whole-brain functional interactions [21]. Functional magnetic resonance imaging (fMRI) provides a useful tool for estimating functional connectivity among brain regions and has been widely used to study large-scale brain network organization [22,23,24]. Compared with resting-state fMRI, task-state fMRI acquired during speech listening can capture functional interactions that are more directly related to auditory speech processing [25,26,27]. Nevertheless, fMRI functional connectivity is indirect, undirected, and correlational; therefore, when it is used to constrain SNN topology, it should be interpreted as a biologically inspired structural prior rather than as a direct model of biological information transmission. In this study, we propose a task-state fMRI-constrained SNN framework for speech recognition. The central idea is that fMRI data acquired during an auditory speech-listening task contain speech-evoked functional connectivity patterns of the human brain. These task-state functional connectivity patterns can be used as a biologically meaningful structural prior for constructing the recurrent topology of an SNN reservoir. Importantly, the proposed framework does not use fMRI signals as direct inputs for speech recognition. Instead, the fMRI data are used offline to extract a task-state brain functional topology, while the speech signal remains the only external input during SNN-based recognition. Because the fMRI data are used only to define a static reservoir topology, no chronological alignment is required between the fMRI time series and the speech samples used for recognition.

The fMRI data used for topology construction were acquired during naturalistic English audiobook listening, whereas the downstream speech recognition experiment was performed on isolated spoken digits. Therefore, the extracted topology is interpreted as a general speech-listening functional prior rather than a digit-specific neural representation. Specifically, the Schaefer-400 cortical parcellation is used to define 400 whole-brain functional nodes. In the proposed framework, all 400 parcels are retained for topology construction, including both auditory and non-auditory brain regions. This design is based on the assumption that speech perception involves distributed whole-brain cooperation, in which non-auditory regions may also support auditory attention, temporal integration, semantic processing, and cross-regional information transmission. Thus, the proposed reservoir is constrained by a 400-node whole-brain task-state functional topology rather than by a small subset of auditory regions only.

Within this 400-node whole-brain topology, 7 SomMotB_Aud parcels are further identified as auditory core nodes. These nodes are mainly located in bilateral auditory cortical and superior temporal auditory-related regions and are used to characterize the auditory core circuit embedded in the whole-brain functional network. It should be noted that these 7 auditory nodes are not the only nodes used for reservoir construction. Instead, they serve as representative auditory core nodes for analyzing whether the speech-listening task activates the auditory processing circuit within the broader whole-brain network. The contribution of this embedded auditory circuit is further examined through dedicated auditory-core analysis.

To support the task relevance of the extracted topology, functional connectivity among the 7 auditory core nodes is compared between the speech-listening task state and the resting state. Compared with resting-state fMRI, the speech-listening task-state fMRI shows stronger correlations among these auditory core nodes, indicating that the auditory processing circuit is activated and functionally strengthened under speech stimulation. This result suggests that the task-state fMRI-derived topology contains speech-evoked auditory functional organization, rather than merely reflecting spontaneous resting-state connectivity. In addition, task-state and resting-state topology-constrained SNNs are compared under the same modeling conditions to examine the task-related contribution of the speech-listening topology.

The extracted 400-node task-state whole-brain functional topology is then mapped onto the recurrent connectivity of the SNN reservoir. Each Schaefer parcel corresponds to one reservoir neuron, and the functional connectivity between brain regions is used to constrain the synaptic connection pattern of the reservoir. This one-parcel-to-one-neuron mapping is a scale-matched computational abstraction that preserves the graph organization of the Schaefer-400 topology, rather than a claim that one cortical parcel is biologically equivalent to one single neuron. Speech signals are converted into spike trains through auditory-inspired preprocessing and spike encoding, and the resulting spike trains drive the SNN dynamics for speech classification. In this way, the proposed framework integrates speech-driven spiking computation with human brain functional organization derived from task-state fMRI.

To evaluate the robustness of the proposed topology, threshold sensitivity testing, resting-state topology comparison, auditory-core contribution analysis, and statistical significance testing are conducted. These analyses examine whether the performance advantage of the proposed model is stable and related to the task-state whole-brain functional topology.

The main contributions of this paper are summarized as follows:

(1) A task-state fMRI-constrained SNN framework is proposed for speech recognition, in which human brain functional connectivity during speech listening is used as a structural prior for reservoir construction. The fMRI-derived topology is used only offline, while speech spike trains remain the only external input during recognition.
(2) Unlike approaches that use only selected auditory regions, the proposed method retains all 400 Schaefer parcels to construct a whole-brain task-state functional topology, thereby preserving distributed cortical interactions involved in speech perception. The 400-node topology is interpreted as a general speech-listening functional prior rather than a digit-specific neural representation.
(3) Within the 400-node whole-brain topology, 7 SomMotB_Aud parcels are identified as auditory core nodes for embedded circuit analysis rather than for reservoir node selection. The enhanced functional connectivity among these auditory nodes during speech listening, compared with the resting state, indicates activation of the auditory core circuit under speech stimulation. The contribution of the embedded auditory circuit is further evaluated through auditory-core analysis.
(4) The proposed topology-constrained SNN provides a biologically interpretable reservoir structure for speech recognition by embedding a speech-activated auditory core circuit within a distributed whole-brain functional network. The fMRI-to-SNN mapping is treated as a topology-constrained computational abstraction, and the robustness of the topology is evaluated using resting-state comparison, threshold-sensitivity analysis, and statistical testing.

The remainder of this paper is organized as follows: Section 2 introduces the proposed method, including task-state fMRI preprocessing, whole-brain functional topology construction, auditory core node analysis, SNN modeling, and the speech recognition framework. Section 3 presents the experimental setup. Section 4 provides the experimental results and discussion. Section 5 concludes the paper.

## 2. Materials and Methods

This study extends the previously established fMRI-SNN framework by replacing the resting-state or generic functional topology with a speech-listening task-state whole-brain functional topology derived from fMRI data. Unlike an auditory-region-only topology, the proposed framework retains all 400 Schaefer cortical parcels as reservoir nodes, while the 7 SomMotB_Aud parcels are analyzed as an embedded auditory core circuit within the whole-brain network. The main objective is to investigate whether speech-listening task-state whole-brain functional connectivity can provide a more biologically meaningful reservoir structure for SNN-based speech recognition, while the embedded auditory core circuit is used to verify task-related auditory activation.

In this study, the proposed model is referred to as the Task-State Whole-Brain fMRI-SNN (TSWB-fMRI-SNN). The core component of the TSWB-fMRI-SNN is an SNN reservoir whose topology is constrained by task-state whole-brain functional connectivity derived from speech-listening fMRI data.

### 2.1. Overall Framework

The proposed framework consists of two main stages: task-state fMRI-based brain topology construction and topology-constrained SNN-based speech recognition. The central assumption of this study is that human fMRI signals acquired during speech listening contain task-modulated functional interactions related to auditory speech processing [26]. In particular, compared with resting-state conditions, speech stimulation is expected to modulate the whole-brain functional topology and, in particular, enhance functional connectivity among the embedded auditory core nodes, indicating the activation of auditory processing circuits.

In the first stage, task-state fMRI data acquired while participants listened to naturalistic speech are used to construct a whole-brain functional topology. The Schaefer-400 cortical parcellation is adopted as the parent atlas, and all 400 cortical parcels are retained as network nodes. Pairwise functional connectivity among the 400 nodes is calculated based on their BOLD time series, resulting in a 400 × 400 task-state functional connectivity matrix.

Within this whole-brain topology, 7 SomMotB_Aud parcels are identified as auditory core nodes. These nodes are mainly located in bilateral auditory cortical and superior temporal auditory-related regions. Rather than being the only nodes used for topology construction, these 7 nodes are used to characterize the auditory core circuit within the whole-brain task-state network. Their enhanced functional connectivity under speech stimulation provides evidence that the auditory processing circuit is activated during the speech-listening task.

In the second stage, speech signals are transformed into spike trains through auditory-inspired preprocessing and spike encoding. These speech spike trains are fed into an SNN reservoir whose recurrent connectivity is constrained by the fMRI-derived 400-node task-state functional topology. The reservoir converts the temporal speech spike trains into dynamic neuronal firing patterns, which are subsequently read out by a classification layer for speech recognition.

It should be emphasized that the fMRI data are not used as direct recognition inputs. Instead, they are used offline to derive a biologically meaningful task-state brain topology prior. During SNN-based speech recognition, the speech signal remains the only external input to the model. In this way, the proposed framework incorporates speech-evoked human brain functional organization into the SNN architecture, allowing the reservoir to preserve both whole-brain functional interactions and task-activated auditory processing circuits.

**Figure 1:**
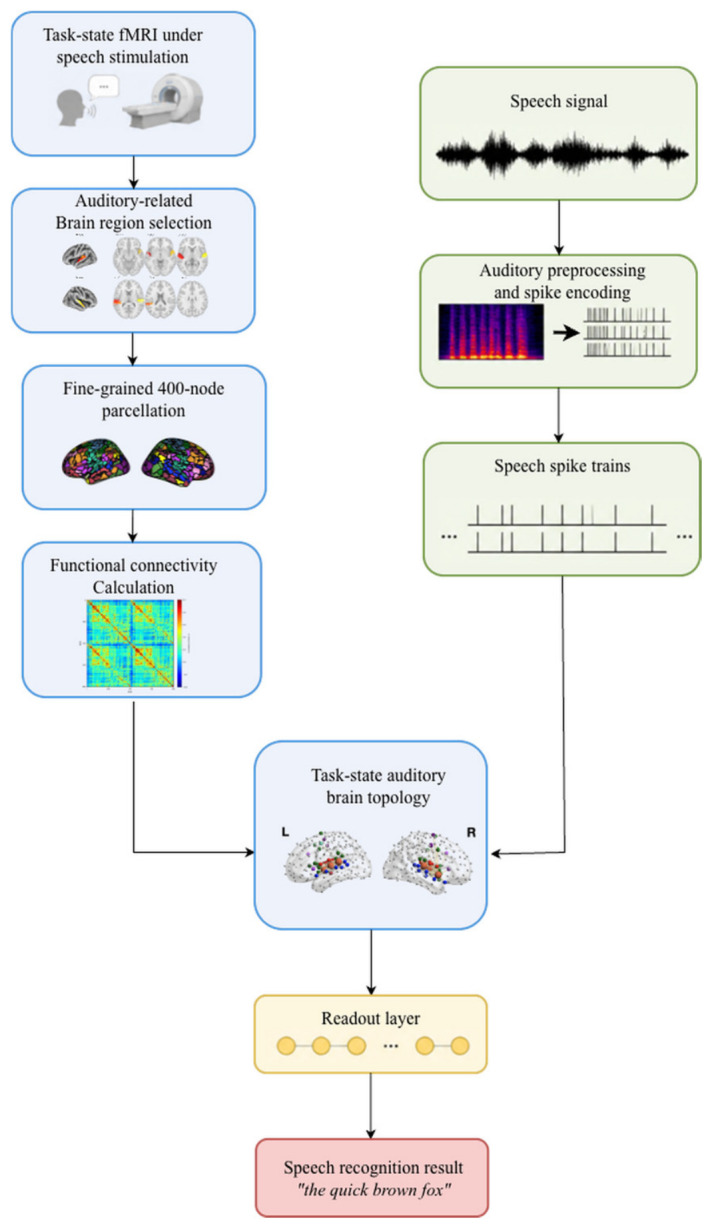
The overall workflow. Arrows indicate the sequential workflow from fMRI-based topology construction to SNN-based speech recognition, and colors distinguish the main processing modules.

The overall workflow is summarized in Figure 1.

### 2.2. Task-State fMRI Data and Whole-Brain Functional Topology Construction

#### 2.2.1. Task-State fMRI Data

In this study, task-state fMRI refers to human BOLD signals recorded during an auditory speech-listening task. Unlike resting-state fMRI, the task-state fMRI data used here were acquired while participants listened to naturalistic English speech. Therefore, the resulting BOLD time series are expected to contain functional responses and inter-regional coordination patterns related to auditory speech perception.

The fMRI data were obtained from the publicly available OpenNeuro ds003643 dataset, also known as the Le Petit Prince fMRI Corpus. Only the English subset was used in this study to minimize potential variability caused by language differences. This subset includes 49 healthy young adult native English speakers, including 30 females, who listened to the English audiobook of The Little Prince during multi-echo fMRI scanning. In the present study, these 49 participants were not averaged into a single group-level functional topology at the preprocessing stage. Instead, each participant was used to construct an individual subject-specific 400-node task-state functional connectivity matrix. The released English subset had been selected after quality control for head motion in the original corpus.

According to the original preprocessing protocol, anatomical images were aligned to the MNI template [28], and functional images underwent removal of the first four volumes, slice-timing correction, despiking, volume registration, nonlinear alignment to MNI space, multi-echo independent component analysis denoising, and resampling to 2 mm isotropic voxels. In the present study, the preprocessed BOLD time series from the English subset were used for node-level time-series extraction, task-state functional connectivity estimation, auditory core node analysis, and SNN reservoir topology construction. For the resting-state control analysis, resting-state fMRI data from the same 49 participants were also used to construct a 400-node whole-brain functional topology under the same Schaefer-400 parcellation. The resting-state topology was generated using the same node-level time-series extraction, Pearson-correlation-based functional connectivity estimation, absolute-value transformation, and thresholding procedure as the task-state topology. The resulting resting-state topology was used only as a control reservoir structure in the task-state versus resting-state comparison, whereas the primary proposed model was constructed from the speech-listening task-state fMRI topology.

Let the preprocessed BOLD signal of the *i*-*th* Schaefer parcel be denoted as:

(1) $$x_{i}\left(t\right),\;i=\;1,\;2,\;\ldots ,\;N_{A},\;t=1,2\ldots T$$

where *t* denotes the time point, *T* denotes the number of time points, and *N* denotes the number of brain network nodes. In this study, *N* = 400, corresponding to the 400 cortical parcels in the Schaefer-400 parcellation.

#### 2.2.2. Schaefer-400 Whole-Brain Node Definition

**Figure 2:**
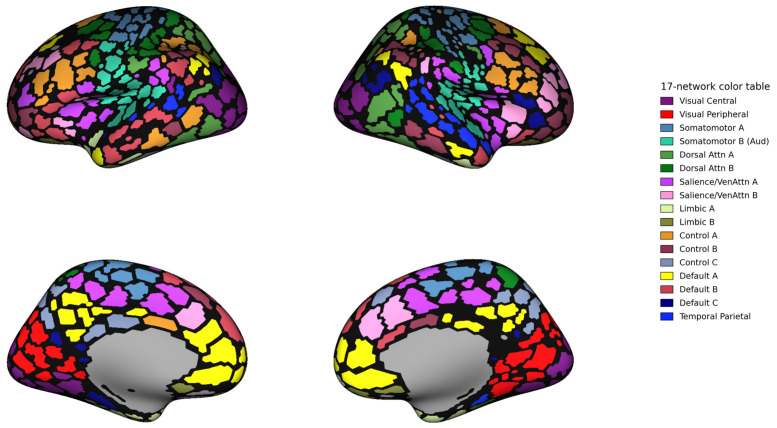
Schaefer-400 whole-brain cortical parcellation used for topology construction. Each parcel represents one cortical functional node, and all 400 parcels are retained as reservoir nodes in the proposed SNN.

To obtain a fine-grained representation of task-state whole-brain functional organization, the Schaefer-400 cortical functional parcellation with 17-network ordering in FSL MNI152 2 mm space was used as the brain atlas. This atlas contains 400 cortical parcels covering the cerebral cortex. In this study, all 400 parcels were retained as functional brain nodes for constructing the task-state fMRI-derived topology, as shown in Figure 2.

The Schaefer-400 atlas provides a whole-brain cortical parcellation covering distributed functional systems, including auditory, sensorimotor, attention, control, temporal, parietal, and frontal cortical regions. In this study, no auditory-region-only selection was performed for topology construction. All 400 cortical parcels were retained as nodes to preserve distributed cortical interactions during speech listening. Auditory-related regions were further examined only through the 7 SomMotB_Aud parcels as an embedded auditory core circuit.

Different from approaches that restrict the reservoir topology to selected auditory regions, this study constructs the reservoir topology from the full 400-node whole-brain task-state functional connectivity matrix. This design preserves both auditory and non-auditory cortical interactions involved in speech listening, while allowing the 7 SomMotB_Aud parcels to be analyzed as an embedded auditory core circuit.

#### 2.2.3. Auditory Core Node Identification

**Figure 3:**
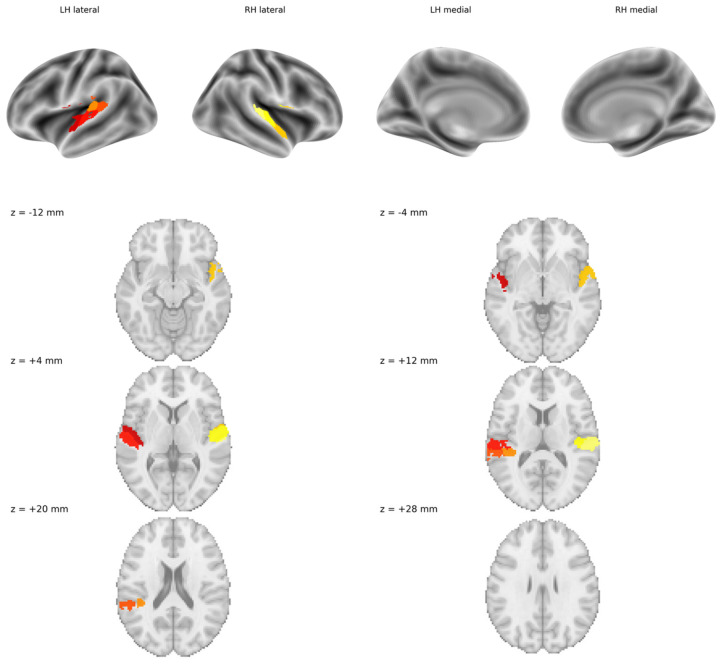
Identification of the embedded auditory core circuit within the Schaefer-400 whole-brain topology. The highlighted SomMotB_Aud parcels are used for auditory-core activation analysis, not as the complete reservoir node set.

Within the 400-node whole-brain topology, 7 SomMotB_Aud parcels were further identified as auditory core nodes. As shown in Figure 3, these auditory core nodes were mainly located in bilateral auditory cortical and superior temporal auditory-related regions. These regions are closely associated with speech sound perception and auditory feature processing, and were therefore used to characterize the core auditory processing circuit activated by speech stimulation.

Importantly, these 7 auditory core nodes were not used to reduce the whole-brain topology or determine the reservoir size. They were used only to evaluate whether the speech-listening task enhanced the auditory core subnetwork embedded in the 400-node whole-brain topology. To further examine whether the embedded auditory core circuit contributed to speech recognition performance, an auditory-core contribution analysis was performed. In this analysis, the full 400-node TSWB-fMRI-SNN was compared with auditory-core control variants. First, an auditory-core-disrupted topology was constructed by removing the recurrent connections within and incident to the 7 SomMotB_Aud parcels while retaining the remaining whole-brain topology. Second, a non-auditory-only topology was constructed by excluding the 7 auditory core nodes and retaining the remaining 393 non-auditory Schaefer parcels as reservoir nodes. The neuron model, synaptic dynamics, speech encoding method, input projection strategy, reservoir-to-output training method, training epochs, and testing protocol were kept identical across different topology conditions. Therefore, the performance differences among these models were used to evaluate the contribution of the embedded auditory core circuit to speech recognition.

#### 2.2.4. Whole-Brain Functional Connectivity and Topology Construction

After defining all 400 Schaefer cortical parcels, the mean BOLD time series of each parcel was extracted by averaging the time series of all voxels within that parcel. Pairwise Pearson correlations were then calculated between all pairs of parcels, resulting in a 400 × 400 whole-brain task-state functional connectivity matrix. Pearson correlation analysis was then used to estimate the functional association between node i and node j:

(2) $$R_{ij}=\frac{\sum_{t=1}^{T}\left(x_{i}\left(t\right)-{\bar{x}}_{i}\right)\left(x_{j}\left(t\right)-{\bar{x}}_{j}\right)}{\sqrt{\sum_{t=1}^{T}{\left(x_{i}\left(t\right)-{\bar{x}}_{i}\right)}^{2}\sum_{t=1}^{T}{\left(x_{j}\left(t\right)-{\bar{x}}_{j}\right)}^{2}}}$$

where $x_{i}\left(t\right)$ and $x_{j}\left(t\right)$ denote the BOLD signals of nodes i and j at time point t, respectively. ${\bar{x}}_{i}$ and ${\bar{x}}_{j}$ are the corresponding mean BOLD signals, and T is the total number of time points.

The functional connectivity strength between two nodes was defined as the absolute value of the Pearson correlation coefficient. In this study, the absolute value was used because the topology construction focused on connection strength rather than the direction of correlation.

(3) $$FC_{ij}=\left|R_{ij}\right|$$

Accordingly, the functional connectivity matrix was obtained as:

(4) $$FC\epsilon R^{N*N}$$

where N=400 represents the number of whole-brain cortical parcels in the Schaefer-400 parcellation. Each element $FC_{ij}$ in the matrix reflects the functional coupling strength between Schaefer parcel i and Schaefer parcel j, and serves as the basis for subsequent whole-brain topology generation and SNN reservoir construction.

**Figure 4:**
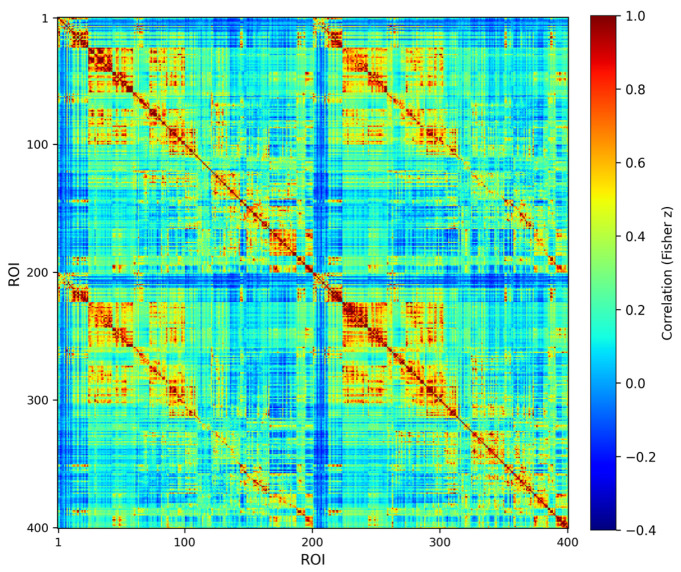
The 400 × 400 whole-brain functional connectivity matrix calculated from speech-listening task-state fMRI signals. Each matrix element represents the Pearson correlation-based functional connectivity strength between two Schaefer cortical parcels.

As illustrated in Figure 4, the above calculation produced a node-by-node functional connectivity matrix.

The color of each matrix element represents the connection strength between two nodes, with warmer colors indicating stronger functional coupling and cooler colors indicating weaker coupling. The diagonal elements correspond to self-connections and were excluded from later topology construction. This procedure follows the graph-theoretical strategy used in fMRI-derived brain network analysis, where brain regions are regarded as nodes and functional connectivity between regions is regarded as edges.

#### 2.2.5. Thresholding and Topology Generation

After obtaining the 400 × 400 whole-brain functional connectivity matrix, a thresholding strategy was applied to generate the sparse task-state whole-brain topology for the SNN reservoir. The purpose of this step was to retain strong task-related whole-brain functional connections while removing weak or noisy correlations. For topology construction in the TSWB-fMRI-SNN, a threshold $X_{th}$ was introduced to binarize the functional connectivity matrix and determine inter-node connections. Node pairs with functional connectivity values greater than $X_{th}$ were regarded as connected, whereas those not exceeding this threshold were considered disconnected.

For each pair of Schaefer parcels i and j, the binary adjacency matrix was defined as:

(5) $$A_{ij}=\left\{\begin{array}{c} 1,\;{FC}_{ij}>\theta \\ 0,\;{FC}_{ij}\leq \theta \end{array}\right.$$

where θ, also denoted as $X_{th}$, represents the functional connectivity threshold. If the connectivity strength between two Schaefer parcels was greater than the threshold θ, an edge was retained between the corresponding parcels; otherwise, no edge was retained.

For the weighted topology, the edge weight was further determined by the corresponding functional connectivity strength:

(6) $$W_{ij}=A_{ij}\times {FC}_{ij}$$

where $W_{ij}$ represents the connection weight between node i and node j. In this way, the generated topology preserved both the structural connection pattern and the relative strength of task-state functional connectivity.

To obtain a biologically plausible and relatively sparse reservoir topology, the threshold was selected by considering the reported density range of biological brain networks and the stability of network sparsity across participants. Instead of arbitrarily fixing the threshold, adjacent threshold values were further examined in the threshold-sensitivity analysis. The detailed density results and recognition performance comparison under different thresholds are presented in Section 4.3. Based on this analysis, $X_{th}\;$= 0.5 was adopted as the functional connectivity threshold in the main experiments.

**Figure 5:**
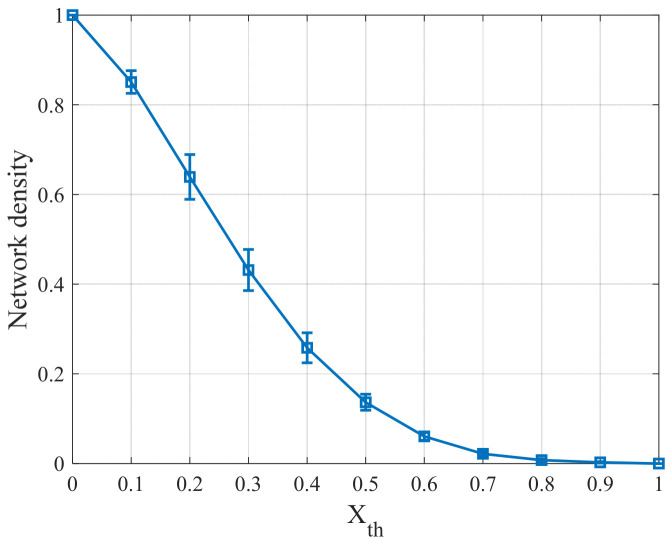
Network density of the 400-node whole-brain task-state topology under different functional connectivity thresholds. Error bars represent the standard deviation across participants.

The relationship between the functional connectivity threshold and the resulting network density is summarized in Figure 5.

**Figure 6:**
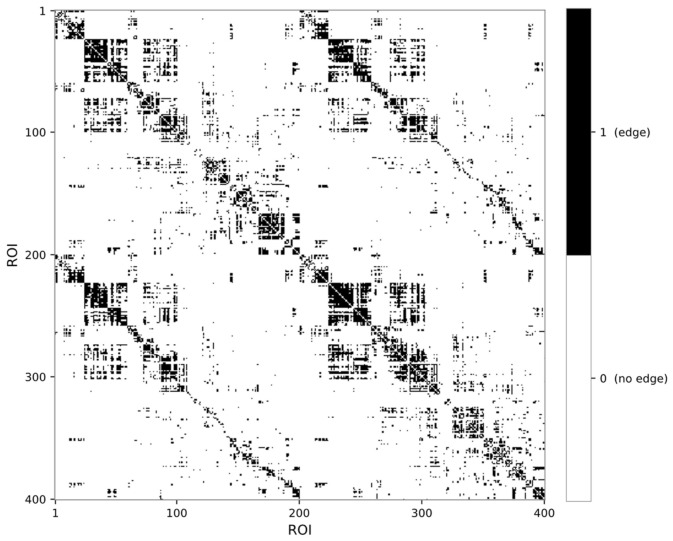
Binary adjacency matrix of the thresholded 400-node whole-brain task-state topology. Black elements indicate retained functional connections between Schaefer parcels, whereas white elements indicate no retained connection.

According to this threshold, the edge connections of the task-state functional brain network were determined. Functional connections with strengths greater than $X_{th}=0.5$ were retained and assigned a value of 1 in the binary adjacency matrix, whereas connections with strengths less than or equal to $X_{th}=0.5$ were removed and assigned a value of 0. The resulting binary edge connection matrix is shown in Figure 6.

Based on this threshold, only relatively strong task-state functional connections were retained, allowing the resulting topology to preserve important speech-related whole-brain functional interactions, including the embedded auditory core circuit, while avoiding excessive weak or noisy connections. Since the functional connectivity matrix was estimated from task-state fMRI data during naturalistic English speech listening, the thresholded topology was regarded as a task-state whole-brain functional topology with an embedded auditory core circuit and was subsequently mapped onto the TSWB-fMRI-SNN reservoir.

For the resting-state control, a 400-node whole-brain functional connectivity matrix was constructed using resting-state fMRI data under the same Schaefer-400 parcellation. The same Pearson-correlation-based functional connectivity estimation, absolute-value transformation, and thresholding strategy were applied to generate the resting-state topology. The threshold was also set to $X_{th}=0.5$, so that the task-state and resting-state topology-constrained SNNs were compared under the same node definition, thresholding criterion, reservoir size, neuron model, synaptic plasticity mechanism, speech encoding method, input projection strategy, training method, and testing protocol.

### 2.3. Spiking Neural Network Model

In the proposed TSWB-fMRI-SNN, the number of reservoir neurons was set equal to the number of Schaefer cortical parcels, namely $N_{r}=400$. Each reservoir neuron corresponded to one Schaefer parcel in the 400-node whole-brain task-state functional topology.

#### 2.3.1. Izhikevich Neuron Model

The Izhikevich neuron model is adopted because it can reproduce various biological firing patterns while maintaining relatively low computational complexity. The model is described as:

(7) $$\left\{\begin{array}{c} dV_{I}/dt=0.04V_{I}^{2}+5V_{I}+140-u+I_{e}\\ du/dt=a(bV_{I}-u)\\ if\;V_{I}\geq 30,\;\left\{\begin{array}{c} V_{I}\leftarrow c\\ u\leftarrow u+d \end{array}\right. \end{array}\right.$$

where $V_{I}$ is the membrane potential of the Izhikevich neuron model; u is the recovery variable for $V_{I}$; $I_{e}$ represents the external input current; a, b, c, and d are dimensionless parameters that can be adjusted to simulate the firing sequences of excitatory and inhibitory Izhikevich neurons, as shown in Table 1.

#### 2.3.2. Excitatory and Inhibitory Neurons

To simulate the excitation–inhibition balance in biological neural systems, neurons in the SNN reservoir are divided into excitatory and inhibitory neurons. The ratio is set as:

(8) $$N_{E}:N_{I}=4:1$$

where $N_{E}$ and $N_{I}$ denote the numbers of excitatory and inhibitory neurons, respectively.

Excitatory neurons promote neural information transmission, while inhibitory neurons regulate firing activity and prevent excessive synchronization. This setting follows the commonly used excitatory–inhibitory proportion in cortical-inspired SNN reservoirs.

#### 2.3.3. Synaptic Model

In this study, a conductance-based synaptic model was used to describe excitatory and inhibitory synaptic interactions in the SNN reservoir [29]. To incorporate the speech-listening task-state whole-brain topology into the SNN, synaptic connections were established only between neuron pairs connected in the thresholded 400-node whole-brain task-state functional topology.

The synaptic current was described as:

(9) $$I_{syn}\left(t\right)=g\left(t\right)r_{g}\left(t\right)\left(E_{syn}-V_{post}\left(t\right)\right)$$

where $I_{syn}$ is the synaptic current; g is the synaptic weight; $E_{syn}$ is the synaptic reversal potential; $V_{post}$ is the postsynaptic membrane potential; $r_{g}$ is the fraction of bound receptors, which reflects changes in concentration of the neurotransmitter H. It can be described as follows:

(10) $$\left\{\begin{array}{c} \frac{dr_{g}}{dt}=\alpha H\left(1-r_{g}\right)-\beta r_{g}\\ H={\left[1+\exp\left(-V_{pre}\left(t-\tau \right)\right)\right]}^{-1} \end{array}\right.$$

where α is the forward rate constant for the neurotransmitter; β is the reverse rate constant for the neurotransmitter; $V_{pre}$ is the presynaptic membrane potential; τ is the synaptic time delay. The excitatory and inhibitory synaptic weights, denoted as $g_{ex}$ and $g_{in}$, respectively, were regulated based on the following update rules [30].

When a postsynaptic neuron j does not receive an action potential from a presynaptic neuron i, the values of $g_{ex}$ and $g_{in}$ decrease exponentially, which can be described as follows:

(11) $$\tau_{ex}\;dg_{ex}/dt=-g_{ex};{\;\tau }_{in}\;dg_{in}/dt=-g_{in}$$

where $\tau_{ex}$ and $\tau_{in}$ are the decay constants for $g_{ex}$ and $g_{in}$, respectively.

When j receives an action potential from i, the values of $g_{ex}$ and $g_{in}$ change, which can be described as follows:

(12) $$\left\{\begin{array}{c} g_{ex}\left(t\right)=g_{ex}\left(t\right)+{\bar{g}}_{ex}\\ {\bar{g}}_{ex}=w\left(∆t\right)*g_{max} \end{array};\;w\left(∆t\right)=\left\{\begin{array}{c} A_{+}\exp\left(∆t/\tau_{+}\right),∆t<0\\ {-A}_{-}\exp\left(∆t/\tau_{-}\right),∆t\geq 0 \end{array}\right.\right.$$

(13) $$\left\{\begin{array}{c} g_{in}\left(t\right)=g_{in}\left(t\right)+{\bar{g}}_{in}\\ {\bar{g}}_{in}=m\left(∆t\right)*g_{max} \end{array}\right.;\;m\left(∆t\right)=\left\{\begin{array}{c} {-B}_{+}\exp\left(∆t/\tau_{+}\right),∆t<0\\ B_{-}\exp\left(∆t/\tau_{-}\right),∆t\geq 0 \end{array}\right.$$

where ${\bar{g}}_{ex}$ and ${\bar{g}}_{in}$ are the increments of $g_{ex}$ and $g_{in}$, respectively; $g_{max}$ is the maximum value of the synaptic weight: if ${g>g}_{max}$, it is set to $g_{max}$, whereas if g<0, it is set to 0; w(∆t) and m(∆t) are the modification functions for the spike-timing-dependent plasticity (STDP) rule; ∆t is the time interval between the presynaptic and postsynaptic spikes; $A_{+}$ and $B_{+}$ are the maximum modification values of increased $g_{ex}$ and $g_{in}$, respectively; $A_{-}$ and $B_{-}$ are the minimum modification values of reduced $g_{ex}$ and $g_{in}$, respectively; $\tau_{+}$ and $\tau_{-}$ are the neuronal firing interval for increased and reduced g, respectively, as shown in Table 2.

#### 2.3.4. Mapping the 400-Node Whole-Brain Functional Topology to the SNN Reservoir

After generating the thresholded 400-node task-state whole-brain topology, the network was mapped onto the recurrent layer of the SNN reservoir. Each Schaefer cortical parcel was mapped to one reservoir neuron, and each retained functional connection between two parcels was mapped to a synaptic connection between the corresponding reservoir neurons. Therefore, the binary adjacency matrix $A\in R^{400\times 400}$ determined the recurrent structural connectivity of the reservoir, and the topology-derived weight matrix $W\in R^{400\times 400}$ was used to initialize the corresponding synaptic weights.

Specifically, if an edge existed between parcel i and parcel j in the thresholded whole-brain topology, namely $A_{ij}=1$, a synaptic connection was established between the corresponding reservoir neurons. If $A_{ij}=0$, no recurrent synaptic connection was established. The 7 SomMotB_Aud parcels were included among these 400 reservoir neurons, but they were not treated as the only reservoir neurons. The initial synaptic weight was defined as:

(14) $$g_{ij}\left(0\right)=\alpha W_{ij}=\alpha A_{ij}FC_{ij}$$

where $g_{ij}\left(0\right)$ denotes the initial synaptic weight from neuron i to neuron j,$\;{FC}_{ij}$ represents the functional connectivity strength between the two corresponding Schaefer parcels, $A_{ij}$ denotes the binary adjacency value, $W_{ij}$ is the topology-derived connection weight, and α is a scaling coefficient used to adjust the functional connectivity value to an appropriate synaptic weight range.

Because the functional connectivity was estimated with Pearson correlation, it only reflects pairwise co-fluctuation and does not provide causal or directional information. Therefore, the FC matrix is treated as an undirected prior. Since the fMRI-derived functional connectivity matrix is symmetric, no directionality was inferred. Each retained undirected functional edge was implemented as bidirectional synaptic connections in the SNN reservoir. The sign and physiological effect of each synapse were determined by the type of the presynaptic neuron, whereas $g_{ij}(0)$ represented the initial connection strength.

Through this mapping, the SNN reservoir directly inherited the structural organization of the task-state whole-brain functional network with an embedded auditory core circuit. As a result, the connection pattern of the reservoir was no longer randomly generated, but constrained by speech-listening task-state whole-brain functional connectivity, while the auditory core circuit was analyzed as an embedded subnetwork. This design enables the SNN reservoir to incorporate a task-state functional topology prior derived from speech-listening fMRI, thereby improving the biological interpretability of the proposed speech recognition model without assuming a causal direction of information flow in the original fMRI connectivity matrix.

### 2.4. Speech Signal Encoding

Since SNNs process information through discrete spike trains, the input speech signals were first transformed into spike sequences before being fed into the reservoir. In this study, an auditory-inspired encoding strategy was adopted. The original speech signal $s\left(t\right)$ was first decomposed into multiple frequency-channel signals using a cochlea-like filter bank:

(15) $$s\left(t\right)\to \left\{s_{1}\left(t\right),\right.s_{2}\left(t\right),\ldots ,s_{M}\left.\left(t\right)\right\}$$

where M denotes the number of frequency channels. This step simulates the frequency decomposition mechanism of the biological auditory system.

Then, each frequency-channel signal was converted into a spike train using a spike encoding algorithm. The encoded multi-channel spike sequence is represented as:

(16) $$S\left(t\right)=\left\{S_{1}\left(t\right),S_{2}\left(t\right),\ldots ,S_{M}\left(t\right)\right\}$$

where $S_{m}(t)$ denotes the spike train corresponding to the m−th frequency channel. Speech segments with stronger acoustic energy generate denser spikes, whereas weaker segments generate sparser spikes. The obtained spike sequences were then used as the input of the proposed SNN reservoir. This preprocessing procedure is consistent with previous SNN-based speech recognition studies, in which analog speech signals are transformed into spike trains before being fed into the reservoir.

### 2.5. Speech Recognition Framework

Based on the encoded speech spike trains obtained in Section 2.4, a reservoir-computing-based speech recognition framework was constructed for the proposed TSWB-fMRI-SNN. The framework consists of three components: an input layer, a TSWB-fMRI-SNN reservoir, and an output layer.

The input layer receives the multi-channel speech spike trains and projects them into the reservoir. To preserve the frequency-specific information of speech signals, each frequency channel is connected to only a subset of reservoir neurons rather than to all neurons. The encoded spike trains are introduced into the reservoir as external input currents, thereby driving the neuronal firing dynamics of the SNN.

The reservoir contains $\left(N_{r}=400\right)$ spiking neurons, each corresponding to one Schaefer cortical parcel. Its recurrent topology is constrained by the thresholded 400-node whole-brain task-state functional connectivity matrix. Specifically, all 400 Schaefer parcels are mapped to spiking neurons, and retained whole-brain functional connections are mapped to recurrent synaptic connections in the reservoir. The 7 SomMotB_Aud parcels are included within the 400-node reservoir but are analyzed only as an embedded auditory core circuit, rather than being treated as the complete reservoir node set. When speech spike trains are fed into the reservoir, different speech categories induce distinct spatiotemporal firing patterns. The full reservoir state at time t is defined as:

(17) $$H\left(t\right)={\left.\left[h_{1\left(t\right)},h_{2\left(t\right)},\ldots ,h_{N_{r}\left(t\right)}\right.\right]}^{T}$$

where $N_{r}=400$ denotes the total number of reservoir neurons, corresponding to the 400 Schaefer cortical parcels. Here, $h_{i\left(t\right)}$ represents the firing state of the *i*-*th* neuron at time t. If the neuron emits a spike at time t, $h_{i\left(t\right)}=1$; otherwise, $h_{i\left(t\right)}=0$.

For the embedded auditory core circuit, the 7 SomMotB_Aud nodes are treated as a subset of the full reservoir. Let

(18) $$𝒜=a_{1},a_{2},\cdots ,a_{N_{A}},N_{A}=7$$

denote the auditory-core node index set. The auditory-core state can be represented as:

(19) $$HA\left(t\right)={\left[ha_{1}\left(t\right),h_{a_{2}}\left(t\right),\ldots ,h_{a_{N_{A}}}\left(t\right)\right]}^{T},N_{A}=7$$

where $HA\left(t\right)$ is used only for auditory-core analysis and does not replace the full reservoir state $H\left(t\right)$. Therefore, the speech recognition output is based on the full 400-node reservoir representation rather than only on the 7 auditory core nodes.

The output layer maps the full reservoir firing patterns to the corresponding speech categories. Each output unit represents one speech category, and the final recognition result is determined according to the output response:

(20) $$\hat{y}=\arg\underset{k}{\max}O_{k}$$

where $\hat{y}$ denotes the predicted speech category, and $O_{k}$ represents the response of the k-th output unit. In this framework, the full reservoir transforms speech spike trains into high-dimensional neuronal firing representations, while the output layer performs the final classification.

The detailed speech dataset, training–testing partition, comparison methods, and evaluation metrics used to validate the proposed framework are presented in Section 3.

## 3. Experimental Setup

To evaluate the effectiveness of the proposed TSWB-fMRI-SNN, speech recognition experiments were conducted on an isolated-word speech dataset. This section introduces the speech dataset, data partition strategy, comparison models, parameter settings, and evaluation metrics. The experimental results are presented and discussed in Section 4.

### 3.1. Speech Signal Preprocessing

The speech recognition experiments were conducted using a subset of the TI46 corpus provided by the Linguistic Data Consortium [31]. The speech subset used in this study consisted of utterances from 16 English speakers, including eight males and eight females. Each speaker pronounced the ten digits from “zero” to “nine” 25 times, resulting in a total of 4000 speech samples. For the recognition experiments, 2400 utterances were used for training, while the remaining 1600 utterances were reserved for testing. The training and testing sets were constructed in a speaker-balanced and digit-balanced manner, so that utterances from each speaker and each digit category were represented in both sets. This design allowed the model to be evaluated under inter-speaker acoustic variability, including differences in vocal characteristics, pronunciation patterns, and speaking styles. However, because explicit dialect labels and cross-dialect evaluation were not performed, this setup does not constitute a direct test of dialect robustness.

To ensure independence between topology construction and speech-recognition evaluation, the OpenNeuro fMRI data were not used as either training or testing samples for the speech recognition task. The TI46 corpus was used only for isolated-word recognition, whereas the OpenNeuro task-state fMRI data were used only to construct the biologically inspired reservoir topology.

**Figure 7:**
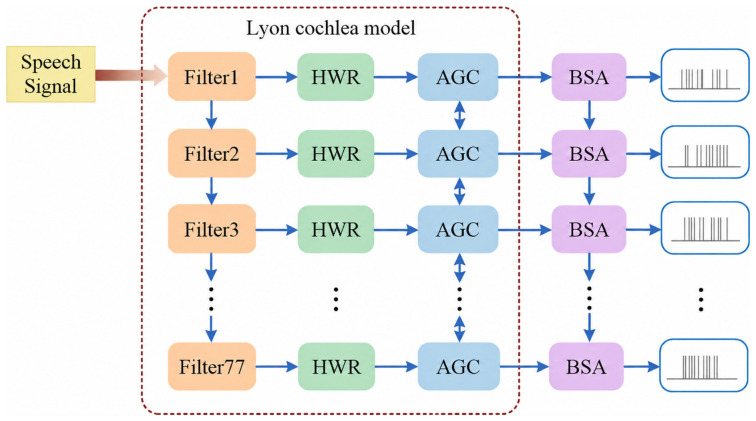
Flowchart of preprocessing for analog speech signals. Arrows indicate the sequential transformation from analog speech waveforms to cochlear-channel responses and spike-train outputs, and colors distinguish the preprocessing and spike-encoding modules.

As SNNs represent and transmit information through discrete spike trains, the continuous speech waveforms were transformed into spike-based representations before being input to the network. In this study, the Lyon cochlea model and Ben’s Spiker Algorithm (BSA) were employed for speech preprocessing and spike encoding, as shown in Figure 7.

(1) Lyon Cochlea Model

The speech signals were processed using the Lyon cochlea model, which consists of 77 cascaded bandpass filters, each extracting a specific frequency band of the speech spectrum. As a result, each speech signal was decomposed into 77 sub-band signals, with the first filter corresponding to the highest-frequency band and the 77th filter corresponding to the lowest-frequency band. The output of each filter was then demodulated using a half-wave rectifier (HWR) and low-pass-filtered to obtain signals within the frequency range relevant to human auditory perception. Finally, automatic gain control (AGC) modules were applied to compress the signal energy and simulate the dynamic-range adaptation of the human auditory system.

(2) Ben’s Spiker Algorithm

Ben’s Spiker Algorithm (BSA) was then used to encode the preprocessed speech signals into spike trains. Speech segments with higher acoustic energy generated denser spike events, whereas segments with lower acoustic energy generated sparser spike events and lower instantaneous peak rates. Through this procedure, each analog speech signal was converted into a multi-channel spike-train representation, which was subsequently used as the input to the proposed task-state whole-brain functional-topology-constrained SNN.

### 3.2. Speech Recognition Framework Based on the Proposed Task-State Whole-Brain fMRI-SNN

**Figure 8:**
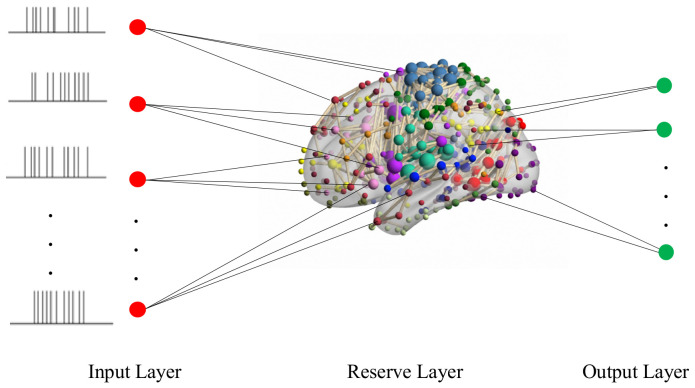
Speech recognition framework based on LSM. Lines indicate information flow among the input layer, TSWB-fMRI-SNN reservoir, and output layer, and colors distinguish the major network components and signal pathways.

Inspired by the liquid state machine (LSM) framework, a speech recognition framework was constructed based on the proposed TSWB-fMRI-SNN. As shown in Figure 8, the framework consisted of an input layer, a task-state whole-brain functional-topology-constrained reservoir, and an output layer.

The input layer receives multi-channel speech spike trains generated from the preprocessed speech signals. The reservoir is implemented as the proposed TSWB-fMRI-SNN, in which each spoken-digit sample evokes a distinct spatiotemporal neuronal firing pattern under the regulation of synaptic dynamics and plasticity. The synaptic weights between the reservoir neurons and the output neurons are trained using the Remote Supervised Method (ReSuMe). During inference, the trained synaptic weights modulate the firing responses of the output neurons, and the class corresponding to the output neuron with the largest spike count is selected as the recognition result.

#### 3.2.1. Input Projection of Speech Spike Trains to the TSWB-fMRI-SNN

In the input layer, the 77 spike trains generated by the Lyon cochlea model and Ben’s Spiker Algorithm (BSA), corresponding to 77 cochlear frequency channels, were randomly projected to a subset of reservoir neurons in the proposed TSWB-fMRI-SNN. Connecting each spike train to all reservoir neurons may weaken the frequency-specific information carried by different cochlear channels. Therefore, the number of reservoir neurons receiving input from each frequency channel may affect the speech recognition performance of the proposed TSWB-fMRI-SNN. To investigate the effect of input projection size, the spike trains from each frequency band were randomly connected to 2, 4, 6, 8, and 10 reservoir neurons in the TSWB-fMRI-SNN, respectively. The resulting recognition accuracies are summarized in Table 3.

As shown in Table 3, the highest recognition accuracy was obtained when each frequency-channel spike train was randomly projected to four reservoir neurons. Therefore, this setting was used in subsequent experiments.

#### 3.2.2. Speech-Evoked Firing Patterns in the Proposed TSWB-fMRI-SNN

**Figure 9:**
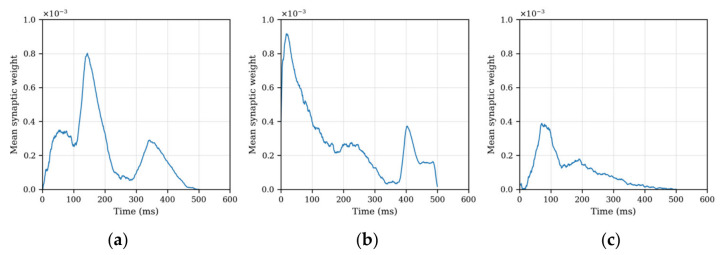
Dynamic changes in mean synaptic weights of TSWB-fMRI-SNN. (**a**) “zero” (**b**) “five” (**c**) “nine”.

The speech spike trains were applied to the Izhikevich neurons in the proposed TSWB-fMRI-SNN as input currents, thereby driving neuronal membrane dynamics and spike responses. During this process, the firing activity of reservoir neurons was adaptively modulated by synaptic dynamics and plasticity mechanisms. To illustrate the synaptic adaptation process driven by speech spike inputs, the temporal evolution of the mean synaptic weights is shown in Figure 9 using the spoken digits “zero”, “five”, and “nine” as representative examples.

**Figure 10:**
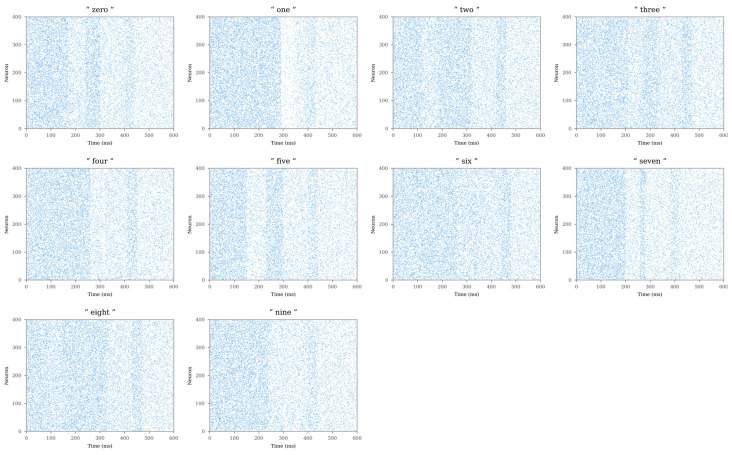
Speech-evoked firing patterns of the proposed TSWB-fMRI-SNN for the ten spoken digits. Each dot represents a spike emitted by a reservoir neuron during the 600 ms simulation window.

As shown in Figure 9, the mean synaptic weights exhibited different temporal trajectories in response to different spoken-digit inputs. According to the neuronal and synaptic dynamic equations, synaptic weights directly influence the input currents and firing responses of reservoir neurons. Consequently, the proposed TSWB-fMRI-SNN generated digit-specific spatiotemporal firing patterns in response to different spoken-digit inputs. The firing pattern in an SNN reflects the dynamic spike responses of reservoir neurons over time. As shown in Figure 10, the proposed TSWB-fMRI-SNN produced distinct firing activities for the spoken digits “zero” through “nine” during the 600 ms simulation period.

As shown in Figure 10, different spoken digits induced distinguishable spatiotemporal firing responses in the proposed TSWB-fMRI-SNN. To statistically evaluate these differences, a one-way ANOVA was performed on the time-resolved population spike counts across the ten digit classes. The results revealed a significant difference among digit-evoked firing patterns $\left(F=6.06,\;\;P=1.64\times 10^{-7}\right)$, confirming that the reservoir generated discriminative neural responses for different speech inputs. Accordingly, these firing patterns were adopted as reservoir features for speech recognition.

#### 3.2.3. Training Process of Synaptic Weights

The synaptic weights between the reservoir neurons and the output neurons were trained using the Remote Supervised Method (ReSuMe) [13]. Inspired by Hebbian learning and spike-timing-dependent plasticity (STDP), ReSuMe is suitable for supervised learning of temporal spike sequences and has been successfully applied to recognition tasks [32,33]. Therefore, ReSuMe was used to update the reservoir-to-output synaptic weight *g*(*t*) as follows [32]:

(21) $$\frac{dg\left(t\right)}{dt}=\left[S\left(t_{d}\right)-S\left(t_{a}\right)\right]\left[e_{H}+\int_{0}^{\infty }w\left(\Delta t\right)S\left(t_{in}-\Delta t\right)dt\right]$$

The parameter e was fixed at 0.25 in this study. Here, $S(t_{in})$ represents the input-induced neuronal spike sequence under speech stimulation, $S(t_{a})$ denotes the actual output spike train after each training epoch, and $S(t_{d})$ indicates the desired output spike train. The desired output sequence was designed according to the digit-specific firing-pattern differences generated by the TSWB-fMRI-SNN.

**Figure 11:**
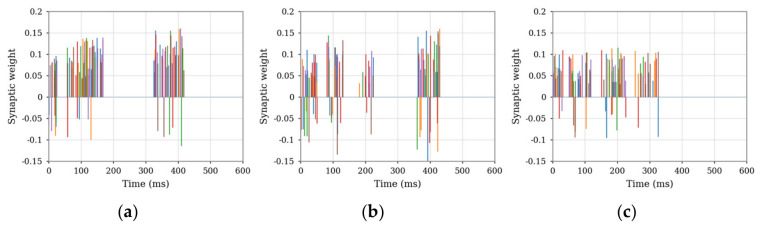
Trained reservoir-to-output synaptic weights for representative spoken digits: (**a**) “zero”, (**b**) “five”, and (**c**) “nine”. Color intensity represents the magnitude of the trained reservoir-to-output synaptic weights, with different colors indicating positive and negative weight components.

During training, one epoch was defined as one complete presentation of all training samples from the ten spoken-digit classes to the proposed TSWB-fMRI-SNN-based speech recognition framework. After 240 training epochs, the reservoir-to-output synaptic weights corresponding to each digit class were obtained. The speech data for the digits “zero”, “five”, and “nine” were taken as examples, and their synaptic weights after training are shown in Figure 11.

As shown in Figure 11, the trained reservoir-to-output synaptic weights exhibited alternating positive and negative components around the desired firing times, thereby promoting target output spikes while suppressing non-target responses.

#### 3.2.4. Decision Results for Speech Recognition

**Figure 12:**
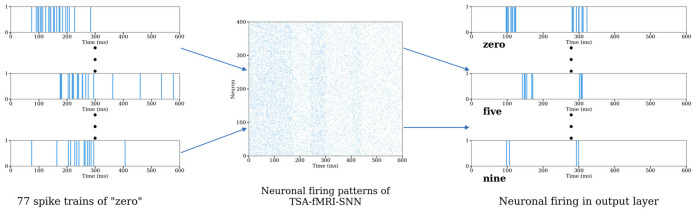
Neuronal firing activities of ‘‘zero’’ during the test process.

During testing, the preprocessed test speech samples were converted into spike trains and input into the proposed TSWB-fMRI-SNN-based speech recognition framework. Driven by the input spike trains and modulated by synaptic dynamics, the reservoir neurons generated speech-specific spatiotemporal firing patterns. The firing activity of the output neurons was modulated by the trained reservoir-to-output synaptic weights. Positive synaptic weights promoted target output firing near the desired firing times, whereas negative synaptic weights suppressed non-target responses. The recognition decision was made according to the firing responses of the output neurons. Specifically, a winner-take-all strategy was adopted, in which the output neuron with the largest spike count was selected as the winning neuron, and its corresponding digit label was assigned as the recognition result. To illustrate the testing process. Figure 12 presents the layer-wise neuronal firing activities for a representative spoken-digit “zero” sample.

The recognition accuracy was defined as the ratio of correctly identified digit utterances to the total number of test utterances. To evaluate the influence of inter-subject variability in the fMRI-derived topology, each of the 49 subject-specific task-state whole-brain topologies was separately mapped onto the SNN reservoir and evaluated using the same speech-recognition protocol. The reported accuracy values were calculated as the mean ± standard deviation across the 49 subject-derived topology conditions. No single group-averaged topology was used as the primary topology for the main recognition results. The proposed TSWB-fMRI-SNN obtained an average accuracy of 95.09 ± 1.01% over repeated trials, and the digit-wise recognition accuracies are summarized in Table 4.

### 3.3. Time Complexity

To examine the computational cost of the proposed TSWB-fMRI-SNN, the speech recognition procedure was analyzed from the perspective of time complexity. The main computations are involved in three parts: updating the reservoir dynamics under speech-driven spike inputs, learning the synaptic weights from the reservoir to the output layer, and producing the final classification decision.

(1) Reservoir dynamic updating

During speech recognition, the encoded speech spike trains are converted into input currents and delivered to the TSWB-fMRI-SNN reservoir. Let p denote the number of input spike trains. The computation associated with the external input current requires O(p) operations. Meanwhile, because each neuron can receive synaptic signals from at most n reservoir neurons, the synaptic-current-related computation has a complexity of O(n).

For k speech samples, each simulated over t time steps, the total computational complexity for updating the firing activities of n reservoir neurons can be written as:

(22) $$O\left(k\times t\times n\times \left(p+n\right)\right).$$

(2) Reservoir-to-output weight learning

The synaptic weights between the reservoir and the output layer are optimized using the ReSuMe learning rule. For a reservoir with n neurons, computing the actual output spike sequence through synaptic plasticity requires $O(n^{2})$ operations, whereas the desired output sequence only contributes only O(1). Since the ReSuMe update also involves temporal integration over t simulation steps, the complexity for one training process is:

(23) $$O\left(\left(1+n^{2}\right)\times t\right)$$

which is dominated by:

(24) $$O\left(n^{2}\times t\right).$$

When m training samples are used and each sample lasts t time steps, the overall training complexity becomes:

(25) $$O\left(m\times t\times n^{2}\times t\right)$$

or equivalently:

(26) $$O\left(m\times n^{2}\times t^{2}\right)$$

(3) Output-layer classification

In the testing stage, the output neurons receive spike-driven signals from the reservoir through n synaptic connections. Therefore, calculating the output-layer firing response requires O(n) operations. For c test samples with a simulation duration of t, the decision-making complexity is:

(27) $$O\left(c\times t\times n\right)$$

Overall, the computational burden of the TSWB-fMRI-SNN mainly comes from reservoir state evolution and ReSuMe-based weight learning. In contrast, the output decision stage requires relatively fewer operations, indicating that the proposed framework remains computationally feasible for the speech recognition task.

### 3.4. Scalability

To evaluate the scalability of the proposed TSWB-fMRI-SNN framework, we measured the reservoir-to-output weight training time under different training-set sizes. The 16-speaker TI46 training set contained 2400 utterances and was divided into five subsets of 480, 960, 1440, 1920, and 2400 utterances. All simulations were performed on a computer equipped with a 2.50 GHz CPU and 8 GB RAM. The corresponding training times are reported in Table 5.

As shown in Table 5, the running time for training the reservoir-to-output synaptic weights increased as the number of utterances increased. However, the increase in running time was smaller than the corresponding increase in the number of utterances, indicating favorable scalability of the proposed method.

### 3.5. Statistical Analysis

To examine whether the performance improvement of the proposed TSWB-fMRI-SNN over the baseline SNN models was statistically significant, statistical analysis was performed on the recognition accuracies obtained from repeated experiments. For each model, the same speech dataset partition, spike encoding procedure, input projection strategy, neuron model, synaptic plasticity mechanism, reservoir-to-output training method, and testing protocol were used. The recognition accuracies obtained under different repeated trials or subject-derived topology conditions were used for statistical comparison.

Before significance testing, the normality of the paired accuracy differences between the proposed TSWB-fMRI-SNN and each comparison model was assessed. If the paired differences satisfied the normality assumption, a paired two-tailed t-test was used; otherwise, the Wilcoxon signed-rank test was adopted. To account for multiple comparisons, the resulting *p*-values were corrected using the false discovery rate (FDR) method. A corrected *p*-value of less than 0.05 was considered statistically significant.

## 4. Results and Discussion

The influence of reservoir topology on recognition performance was examined by comparing the proposed TSWB-fMRI-SNN with SNNs based on regular, random, small-world, scale-free, hybrid small-world/scale-free, and grid structures. To further demonstrate the effectiveness of the proposed framework, its recognition accuracy was also compared with that of previously published speech recognition methods.

### 4.1. Effect of Reservoir Topology on Speech Recognition Performance

In complex network analysis, small-world and scale-free networks are commonly used to characterize complex topological organizations, whereas regular and random networks are often used as reference topologies [34]. Accordingly, regular SNN, random SNN, small-world SNN (sw-SNN), scale-free SNN (sf-SNN), and hybrid small-world/scale-free SNN (swsf-SNN) models were constructed as baseline models. Because randomly connected grid-like reservoirs are commonly used in liquid state machine (LSM) models, a grid-SNN was also constructed for comparison.

#### 4.1.1. Construction of Baseline SNNs with Different Reservoir Topologies

To evaluate the influence of reservoir topology on speech recognition performance, six baseline SNN reservoirs were constructed and compared with the proposed TSWB-fMRI-SNN. To ensure a fair comparison, all baseline SNNs contained the same number of reservoir neurons as the proposed model. Specifically, each reservoir consisted of N=400 spiking neurons, corresponding to the 400 Schaefer parcels used in the proposed task-state fMRI-derived topology. The neuron model, synaptic plasticity mechanism, speech spike encoding method, input projection strategy, reservoir-to-output training method, and decision rule were kept identical across all models. Therefore, the only difference among these SNNs was the recurrent reservoir topology.

**Figure 13:**
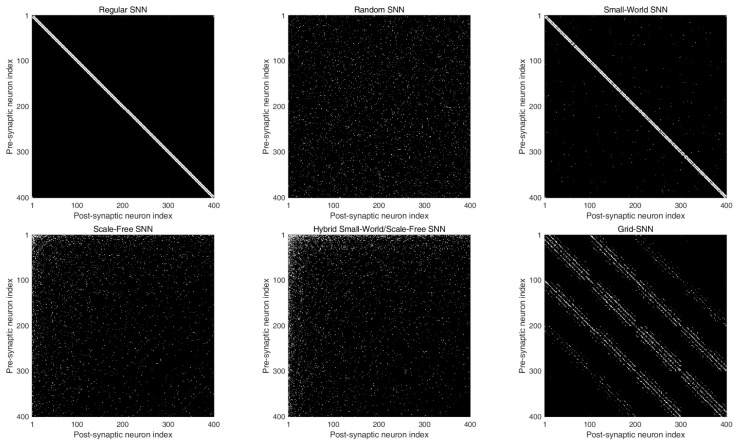
Binary recurrent connection matrices of the six baseline SNN reservoir topologies.

The binary connection matrices of the six baseline reservoir topologies are shown in Figure 13, where the horizontal axis denotes the postsynaptic neuron index and the vertical axis denotes the presynaptic neuron index. White pixels indicate retained synaptic connections, whereas black pixels indicate absent connections. The baseline topologies were generated as described below.

(1) **Regular SNN, Random SNN, and Small-World SNN**

The regular SNN, random SNN, and small-world SNN were generated using the Watts–Strogatz (WS) network model. In this model, an initial ring lattice with N=400 nodes was first constructed, and each node was connected to its nearest neighbors. Then, each edge was rewired with probability p, producing different reservoir topologies by changing the rewiring probability.

For the regular SNN, the rewiring probability was set to p=0. In this case, no edges were rewired, and the resulting reservoir preserved a highly ordered ring-lattice structure. As shown in Figure 13, the corresponding binary topology matrix exhibits clear diagonal band-like patterns, indicating local and regular neighborhood connections.

For the random SNN, the rewiring probability was set to p=1. Under this condition, the original regular connections were fully rewired, resulting in a randomized WS topology. As shown in Figure 13, the connection matrix shows a randomly distributed pattern without an obvious local structure.

For the small-world SNN, the WS rewiring probability was set to p=0.08. This setting preserved part of the local neighborhood structure while introducing a small number of long-range shortcut connections. As shown in Figure 13, the small-world topology retains the diagonal connection bands of the regular network and also contains sparse off-diagonal connections, reflecting a mixture of local clustering and long-range communication.

(2) **Scale-Free SNN**

The scale-free SNN was generated using the Barabási–Albert (BA) preferential attachment model. Starting from a small initial connected network, new nodes were added sequentially until the reservoir size reached N=400. Each newly added node was connected to existing nodes according to the preferential attachment rule, in which nodes with larger degrees had higher probabilities of receiving new connections.

This mechanism produces a heterogeneous degree distribution, where a small number of hub neurons have many synaptic connections and most neurons have relatively few connections. As shown in Figure 13, the topology matrix of the scale-free SNN presents a nonuniform connection distribution, indicating the presence of hub-like nodes formed through preferential attachment.

(3) **Hybrid Small-World/Scale-Free SNN**

The hybrid small-world/scale-free SNN was constructed using a simplified BBV/triadic-closure hybrid mechanism. This topology generation process combined preferential attachment with local triadic closure. Specifically, new nodes were preferentially connected to existing high-degree or high-strength nodes, which introduced scale-free characteristics. Meanwhile, additional local edges were formed through a triadic-closure mechanism, which enhanced local clustering and introduced small-world-like properties.

As shown in Figure 13, the hybrid topology matrix exhibits both heterogeneous connection distribution and locally clustered connection patterns. Therefore, this baseline provides an artificial complex-network topology that combines scale-free hub organization and small-world local clustering, but it is not directly derived from task-state fMRI functional connectivity.

(4) **Grid-SNN**

The grid-SNN was generated using a three-dimensional grid structure with probabilistic distance-dependent connections. To match the reservoir size of the proposed TSWB-fMRI-SNN, a 10 × 10 × 4 grid was constructed, yielding a total of 400 reservoir neurons. Each neuron was assigned a spatial coordinate in the three-dimensional grid, and the connection probability between neuron i and neuron j was determined according to their Euclidean distance D(i,j):

(28) $$Pc\left(i,\;j\right)=\;h\;e^{-\frac{D{\left(i,j\right)}^{2}}{n}}$$

where m controls the distance–decay rate and h controls the overall connection probability scale. Following the common setting in grid-based LSM reservoirs, m was set to 2, and h was specified according to the types of pre- and postsynaptic neurons. Therefore, neurons located closer to each other in the three-dimensional grid had a higher probability of being connected, whereas distant neurons had a lower connection probability.

As shown in Figure 13, the grid-SNN topology matrix displays structured diagonal-like connection patterns caused by spatially local connectivity in the three-dimensional grid. In this way, the grid-SNN provided a conventional LSM-style reservoir baseline with the same reservoir size as the proposed TSWB-fMRI-SNN, while differing only in the recurrent connection topology.

Overall, the above six baseline SNNs all contained 400 reservoir neurons and used the same neuronal, synaptic, encoding, input, and output-training settings as the proposed TSWB-fMRI-SNN. Therefore, their comparison with the proposed model allowed the effect of reservoir topology on speech recognition performance to be evaluated under controlled conditions.

#### 4.1.2. Comparison of Speech Recognition Accuracy

To compare the recognition accuracy of these SNN models, speech recognition experiments were performed using the procedure described in Section 3. From the perspective of network topology, the sw-SNN, sf-SNN, swsf-SNN, and proposed TSWB-fMRI-SNN can be regarded as complex-topology SNNs, whereas the regular SNN and random SNN were used as reference topologies. The grid-SNN was included as a commonly used LSM reservoir baseline. The proposed TSWB-fMRI-SNN was constructed from speech-listening task-state whole-brain functional connectivity, with the 7 SomMotB_Aud parcels being analyzed as an embedded auditory core circuit. By comparison, the swsf-SNN was artificially generated by matching selected topological metrics of the proposed brain-derived functional network. Therefore, the proposed model represents a brain-derived topology-constrained SNN, whereas the swsf-SNN serves as a topology-matched brain-inspired baseline. In contrast, the regular SNN, random SNN, sw-SNN, sf-SNN, and grid-SNN were not directly derived from task-state whole-brain functional connectivity and were therefore used as non-brain-derived baseline models. The recognition accuracies of the regular SNN, random SNN, sw-SNN, sf-SNN, swsf-SNN, grid-SNN, and the proposed TSWB-fMRI-SNN are shown in Table 6.

As shown in Table 6, the complex-topology SNNs, including the proposed TSWB-fMRI-SNN, swsf-SNN, sw-SNN, and sf-SNN, generally achieved higher recognition accuracies than the regular and random SNN baselines. More importantly, the proposed brain-derived speech-listening task-state whole-brain topology achieved the highest recognition accuracy among all compared reservoir topologies. This suggests that preserving distributed whole-brain interactions, while embedding a speech-activated auditory core circuit, provides a more effective topology prior than either artificial topologies or auditory-region-only interpretations.

#### 4.1.3. Statistical Significance Analysis of Recognition Accuracy

To further determine whether the recognition performance improvement of the proposed TSWB-fMRI-SNN was statistically significant, paired statistical comparisons were conducted between the proposed model and each baseline SNN model. In this study, the repeated experiments refer to the 49 subject-specific topology conditions derived from the 49 fMRI participants. For each participant, one task-state whole-brain functional topology was constructed and mapped onto the SNN reservoir, yielding one recognition-accuracy value for the proposed TSWB-fMRI-SNN. To ensure paired comparisons, each baseline SNN model was evaluated under the same speech dataset partition, spike encoding procedure, input projection setting, neuron model, synaptic plasticity mechanism, reservoir-to-output training method, and testing protocol, resulting in 49 matched recognition-accuracy values for each baseline model. Therefore, each statistical comparison was performed using 49 paired accuracy observations. The Wilcoxon signed-rank test was adopted because it does not require the paired accuracy differences to follow a normal distribution and is therefore suitable for the subject-level paired accuracy comparisons in this study. The resulting *p*-values were corrected using the false discovery rate (FDR) method to account for multiple comparisons.

As shown in Table 7, the proposed TSWB-fMRI-SNN achieved higher recognition accuracy than all baseline SNN models. Compared with the regular SNN, random SNN, sw-SNN, sf-SNN, swsf-SNN, and grid-SNN, the proposed model improved recognition accuracy by 8.65, 11.21, 2.59, 4.03, 1.71, and 5.78 percentage points, respectively. After FDR correction for multiple comparisons, all performance improvements remained statistically significant. These results indicate that the performance advantage of the proposed TSWB-fMRI-SNN was not only reflected in the average recognition accuracy but was also supported by statistical significance testing. Therefore, the speech-listening task-state whole-brain functional topology provided a more effective reservoir structure for SNN-based speech recognition than conventional artificial reservoir topologies.

#### 4.1.4. Auditory-Core Contribution Analysis

To examine whether the embedded auditory core circuit contributed to the recognition performance of the proposed model, an auditory-core contribution analysis was conducted. The full 400-node TSWB-fMRI-SNN was compared with two control variants: an auditory-core-disrupted model and a non-auditory-only model. In the auditory-core-disrupted model, the recurrent connections within and involving the 7 SomMotB_Aud parcels were removed while the remaining whole-brain topology was retained. In the non-auditory-only model, the 7 auditory core nodes were excluded, and the remaining 393 non-auditory Schaefer parcels were used to construct the reservoir. All other model settings, including the neuron model, synaptic plasticity mechanism, speech encoding method, input projection strategy, training method, and testing protocol, were kept unchanged.

As shown in Table 8, disrupting the auditory-core-related recurrent connections or removing the auditory core nodes significantly reduced the recognition accuracy compared with the full TSWB-fMRI-SNN. The auditory-core-disrupted model retained the same reservoir size of 400 neurons but removed recurrent connections within and incident to the 7 SomMotB_Aud nodes, resulting in an accuracy decrease from 95.09 ± 0.28% to 93.74 ± 0.36% (FDR-corrected *p* < 0.001). This result indicates that the connectivity pattern involving the embedded auditory core circuit contributed significantly to the recognition performance. In addition, the non-auditory-only model, which excluded the 7 auditory core nodes and retained the remaining 393 Schaefer parcels, achieved an accuracy of 93.12 ± 0.43%, corresponding to a 1.97 percentage-point decrease compared with the full model (FDR-corrected *p* < 0.001). These results suggest that the performance advantage of the proposed TSWB-fMRI-SNN was not produced by non-auditory regions alone, but was associated with embedding the auditory core circuit within the broader task-state whole-brain topology.

#### 4.1.5. Task-State Versus Resting-State Topology Comparison

To further evaluate whether the speech-listening task-state topology provided an advantage over a general spontaneous functional topology, a resting-state topology-constrained SNN was constructed as a control model. The resting-state fMRI-SNN used the same 400 Schaefer parcels as reservoir nodes and was constructed using the same functional connectivity estimation, thresholding strategy, neuron model, synaptic plasticity mechanism, speech encoding method, input projection strategy, reservoir-to-output training method, and testing protocol as the proposed TSWB-fMRI-SNN. The only difference between the two models was that the recurrent reservoir topology was derived from resting-state fMRI rather than speech-listening task-state fMRI.

As shown in Table 9, the task-state TSWB-fMRI-SNN achieved higher recognition accuracy than the resting-state fMRI-SNN. The resting-state fMRI-SNN obtained an accuracy of 93.86 ± 0.37%, whereas the task-state TSWB-fMRI-SNN achieved an accuracy of 95.09 ± 0.28%, corresponding to an improvement of 1.23 percentage points. A paired statistical comparison was conducted across the 49 subject-specific topology conditions. The Wilcoxon signed-rank test was used, and the resulting *p*-value was corrected using the false discovery rate (FDR) method. The task-state TSWB-fMRI-SNN significantly outperformed the resting-state fMRI-SNN (FDR-corrected (*p* < 0.001)). This result suggests that speech-listening task-state functional topology provides a more task-relevant reservoir prior than resting-state functional connectivity.

### 4.2. Comparison with Previously Reported Speech Recognition Methods

In Section 4.1, a controlled comparison was performed in which the reservoir size, neuron model, synaptic plasticity mechanism, speech encoding method, input projection strategy, and output training method were kept identical, while only the reservoir topology was varied. To further evaluate the performance of the proposed TSWB-fMRI-SNN, we compared it with several previously reported speech recognition methods on the TI46 dataset. However, a fully controlled comparison with these studies is difficult because the reported methods differ in learning algorithms, implementation details, data subsets, and training–testing protocols. Therefore, speech recognition accuracy was used as the primary performance metric for an overall benchmark comparison. The methods reported in previous studies [35,36,37,38,39] were selected for comparison because they also used the TI46 speech dataset. The study in [40] used a 400-utterance subset consisting of eight speakers, ten digits, and five utterances per digit, whereas the study in [17] used a 4000-utterance subset consisting of 16 speakers, ten digits, and 25 utterances per digit. To improve comparability with these reported studies, we evaluated the proposed method on five-speaker, eight-speaker, and 16-speaker TI46 subsets. In the five-speaker subset, 300 utterances were used for training, and the remaining utterances were used for testing; in the eight-speaker subset, 200 utterances were used for training, and the remaining utterances were used for testing; and in the 16-speaker subset, 2400 utterances were used for training, and the remaining utterances were used for testing. The recognition accuracies of the proposed method and the previously reported methods are summarized in Table 10.

As shown in Table 10, the proposed method achieved higher recognition accuracy than the previously reported methods under the corresponding TI46 subset settings. This result suggests that the proposed method maintains strong recognition performance when the number of speakers and inter-speaker variability increase. The results in Table 10 also suggest that the proposed model maintained stable performance across the five-speaker, eight-speaker, and 16-speaker subsets with independent training and test samples, indicating robustness across different dataset sizes.

These results also provide evidence for the robustness of the proposed TSWB-fMRI-SNN under inter-speaker variability. In the TI46 experiments, the same fMRI-derived reservoir topology and the same speech encoding strategy were used for all speakers, without introducing speaker-specific topology reconstruction or speaker-dependent parameter tuning. Therefore, the recognition performance across the five-speaker, eight-speaker, and 16-speaker subsets suggests that the proposed topology-constrained SNN can process speech samples with different speaker-dependent acoustic characteristics. However, because the TI46 subset used in this study does not provide explicit dialect labels, dialect-specific generalization was not independently evaluated. Thus, the current results mainly support robustness to speaker variation rather than a definitive conclusion about dialect variation.

The results in Table 10 also indicate that the proposed model has good generalization ability, as it maintained stable recognition performance across the 5-speaker, eight-speaker, and 16-speaker subsets with independent training and test samples. To further examine cross-subset robustness, the reservoir-to-output synaptic weights were trained using the 16-speaker training set and then evaluated on the five-speaker test set. The proposed method achieved an average recognition accuracy of 98.12 ± 0.58% across the five tested speakers, further supporting its robustness under different TI46 subset settings.

Therefore, the proposed model not only performs speech recognition but also provides a biologically inspired computational framework for investigating how speech-listening task-state whole-brain functional organization, together with the embedded auditory core circuit, may support speech-related information processing.

Nevertheless, the current study has several limitations. First, the fMRI-derived topology was obtained from naturalistic audiobook listening, whereas the downstream speech recognition task was performed using isolated spoken digits from the TI46 dataset. Therefore, the resulting topology should be interpreted as a general English speech-listening and language-comprehension functional topology rather than a digit-specific neural representation. In addition, although the TI46 experiments included multiple speakers and therefore allowed inter-speaker variability to be evaluated, explicit dialect labels were not available in the dataset. Consequently, dialect-specific robustness was not independently assessed in the present study and should be further examined using speech datasets with controlled accent or dialect annotations. Second, although the present study constructed subject-level 400-node whole-brain task-state topologies from 49 fMRI participants and analyzed the embedded 7-node auditory core circuit, inter-subject variability in whole-brain topology and auditory-core activation was not examined in depth. Future work should include matched fMRI paradigms using isolated spoken digits, resting-state whole-brain topology controls, auditory-core ablation analyses, and subject-specific topology analyses to further evaluate task specificity and individual variability.

### 4.3. Threshold Sensitivity Analysis

To further examine whether the performance of the proposed model depended heavily on the selected functional connectivity threshold, threshold sensitivity was evaluated by repeating topology construction and speech recognition under adjacent threshold values. This analysis was conducted to justify the threshold selection used in Section 2.2.5 and to test whether the proposed TSWB-fMRI-SNN remained stable when the sparsity of the fMRI-derived topology changed.

First, the network density of the thresholded 400-node whole-brain task-state topology was evaluated under $X_{th}$ = 0.3, 0.4, 0.5, and 0.6. Network density was defined as the ratio of the number of retained edges to the total number of possible edges in the network. The average density and standard deviation were calculated across the 49 subject-specific task-state topologies. As shown in Figure 5, the network density decreased monotonically as the threshold increased. Specifically, the network densities were approximately 0.43 ± 0.04, 0.26 ± 0.03, 0.13 ± 0.02, and 0.06 ± 0.01 at $X_{th}$ = 0.3, 0.4, 0.5, and 0.6, respectively.

Previous biological network studies have reported that the density of biological brain networks is approximately 3.6–39.3% [41]. Therefore, $X_{th}$ = 0.3 produced a relatively dense topology above this biologically plausible range and was not included in the subsequent recognition performance comparison. In contrast, $X_{th}$ = 0.4, 0.5, and 0.6 generated sparse topologies within the reported biological density range and were therefore used for threshold-sensitivity testing.

The task-state whole-brain topology was reconstructed using $X_{th}$ = 0.4, 0.5, and 0.6, and the corresponding TSWB-fMRI-SNN models were evaluated under the same speech-recognition protocol. The neuron model, synaptic plasticity mechanism, speech encoding method, input projection strategy, reservoir-to-output training method, training–testing partition, and testing procedure were kept identical across different threshold conditions.

The recognition accuracies under different thresholds are summarized in Table 11. The proposed model achieved recognition accuracies of 94.62%, 95.09%, and 94.18% at $X_{th}$ = 0.4, 0.5, and 0.6, respectively. Although all three threshold values produced relatively high recognition performance, $X_{th}$ = 0.5 achieved the highest accuracy while maintaining a sparse and biologically plausible network density. Compared with $X_{th}$ = 0.4, 0.5 produced a lower density and smaller inter-subject density variation, indicating more stable sparsity across participants. Compared with $X_{th}$ = 0.6, $X_{th}$ = 0.5 retained more task-state functional connections and avoided excessive removal of potentially relevant speech-related interactions.

These results indicate that the proposed TSWB-fMRI-SNN did not depend on a single arbitrary threshold. Instead, the model maintained stable recognition performance under adjacent biologically plausible thresholds. Therefore, $X_{th}$ = 0.5 was selected as the main threshold because it provided a reasonable balance among biological sparsity, inter-subject stability, and recognition performance.

## 5. Conclusions

In this study, we proposed a task-state whole-brain fMRI-constrained spiking neural network framework for speech recognition. Human fMRI data acquired during naturalistic speech listening were used to construct a 400-node whole-brain functional topology based on the Schaefer-400 parcellation. All 400 cortical parcels were retained as reservoir nodes, rather than using only auditory regions. Within this whole-brain topology, 7 SomMotB_Aud parcels were identified as auditory core nodes to characterize the auditory processing circuit embedded in the broader brain network. Compared with the resting state, the speech-listening task state showed enhanced functional connectivity among the 7 auditory core nodes, indicating that the auditory circuit was activated and functionally strengthened under speech stimulation. The resulting 400-node task-state whole-brain topology was then mapped onto the recurrent connectivity of the SNN reservoir. During recognition, speech spike trains served as the only external input, whereas fMRI data were used only offline for topology construction.

Experimental results showed that the proposed TSWB-fMRI-SNN achieved better speech recognition performance than SNNs with conventional artificial topologies. These findings suggest that task-state whole-brain functional connectivity, together with the activation of auditory core circuits, can provide an effective and biologically interpretable topology prior for SNN-based speech recognition. Future work will further investigate subject-specific topology construction, matched speech-task fMRI paradigms, and adaptive mapping strategies between brain functional networks and large-scale SNN reservoirs.

## Figures and Tables

**Table 1:** Parameters of the Izhikevich neuron model.

Parameter	Description	Value
a	Time scale for u	Excitement: 0.02
		Inhibition: 0.02
b	Sensitivity of *u* to sub-threshold fluctuations of v	Excitement: 0.20
		Inhibition: 0.25
c	Reset value of v	Excitement: −65
		Inhibition: −65
d	Reset value of u	Excitement: 8
		Inhibition: 2

**Table 2:** Parameters of the synaptic plasticity model.

Parameter	Description	Value
E [26]	Reversible synaptic potential	Excitement: 0 mV
		Inhibition: −70 mV
α [26]	Forward rate constant of neurotransmitter	Excitement: 2
		Inhibition: 0.9
β [26]	Reverse rate constant of neurotransmitter	Excitement: 1
		Inhibition: 0.1
$\mu_{ex}$ [26]	Decay constant of the excitatory synaptic weight	3 ms
$\mu_{in}$ [26]	Decay constant of the inhibitory synaptic weight	5 ms
$g_{max}$ [26]	Maximum value of the synaptic weight	0.015
$A_{+}$ [26]	Maximum correction value when the excitatory synaptic weight is increased	0.1
$A_{-}$ [26]	Minimum correction value when the excitatory synaptic weight is reduced	0.105
$B_{+}$ [26]	Maximum correction value when the inhibitory synapse weight is increased	0.02
$B_{-}$ [26]	Minimum correction value when the inhibitory synapse weight is reduced	0.03
$t_{+}$ [26]	Interval range for the presynaptic and postsynaptic firing of neurons when the synaptic weights are increased	20 ms
$t_{-}$ [26]	Interval range for the presynaptic and postsynaptic firing of neurons when the synaptic weights are reduced	20 ms

**Table 3:** Recognition accuracy of the TSWB-fMRI-SNN under different numbers of reservoir neurons receiving input from each frequency channel.

Number of neurons	2	4	6	8	10
Accuracy (%)	90.46	94.68	93.43	89.98	86.32

**Table 4:** Digit-wise recognition accuracy of the proposed TSWB-fMRI-SNN across 49 subject-specific task-state whole-brain topologies.

Digit	Zero	One	Two	Three	Four	Five	Six	Seven	Eight	Nine
Accuracy (%)	98.32 ± 0.53	96.83 ± 1.32	96.67 ± 0.62	89.88 ± 0.33	86.52 ± 0.34	96.42 ± 2.18	95.08 ± 1.96	97.92 ± 0.26	96.88 ± 1.41	96.35 ± 1.11

**Table 5:** Running time of the training synaptic weights.

Data	480	960	1440	1920	2400
Running time (s)	63.58	119.21	176.34	232.43	288.12

**Table 6:** Speech recognition accuracy of SNNs with different topologies.

Network	Topology	Accuracy
regular SNN	regular topology	86.44%
random SNN	random topology	83.88%
sw-SNN	small-world topology	92.50%
sf-SNN	scale-free topology	91.06%
swsf-SNN	small-world and scale-free topology	93.38%
grid-SNN	10 × 10 × 4 grid topology	89.31%
TSWB-fMRI-SNN	400-node task-state whole-brain functional topology	95.09 ± **1.01**%

**Table 7:** Statistical significance analysis between the proposed TSWB-fMRI-SNN and baseline SNN models.

Comparison	Accuracy Difference	FDR-Corrected *p*-Value	Significance
TSWB-fMRI-SNN vs. regular SNN	+8.65%	(*p* < 0.001)	Significant
TSWB-fMRI-SNN vs. random SNN	+11.21%	(*p* < 0.001)	Significant
TSWB-fMRI-SNN vs. sw-SNN	+2.59%	(*p* = 0.006)	Significant
TSWB-fMRI-SNN vs. sf-SNN	+4.03%	(*p* < 0.001)	Significant
TSWB-fMRI-SNN vs. swsf-SNN	+1.71%	(*p* = 0.018)	Significant
TSWB-fMRI-SNN vs. grid-SNN	+5.78%	(*p* < 0.001)	Significant

**Table 8:** Auditory-core contribution analysis of the proposed TSWB-fMRI-SNN.

Model	Reservoir Topology	Reservoir Size	Accuracy	Accuracy Decrease	FDR-Corrected *p*-Value	Conclusion
Full TSWB-fMRI-SNN	400-node task-state whole-brain topology with embedded auditory core circuit	400	**95.09 ± 0.28%**	**--**	**--**	**Reference model**
Auditory-core-disrupted TSWB-fMRI-SNN	400-node topology with recurrent connections involving the 7 auditory core nodes removed	400	**93.74 ± 0.36%**	**−1.35**	***p* < 0.001**	**Significant**
Non-auditory-only TSWB-fMRI-SNN	393-node topology after excluding the 7 auditory core nodes	393	**93.12 ± 0.43%**	**−1.97**	***p* < 0.001**	**Significant**

**Table 9:** Comparison between task-state and resting-state fMRI-derived topology-constrained SNNs.

Model	Topology Source	Reservoir Topology	Accuracy	Accuracy Difference	FDR-Corrected *p*-Value	Conclusion
Resting-state fMRI-SNN	Resting-state fMRI	400-node resting-state whole-brain topology	**93.86 ± 0.37%**	**--**	**--**	**Control model**
Task-state TSWB-fMRI-SNN	Speech-listening task-state fMRI	400-node task-state whole-brain topology with embedded auditory core circuit	**95.09 ± 0.28%**	**+1.23**	***p* < 0.001**	**Significant**

**Table 10:** Comparison with reported methods.

Network	No. of Speakers	Accuracy
SWTA-SNN [32]	8	95.25%
SNN with ST-DFA [33]	16	84.88%
LSM [38]	5	97.50%
LSM [34]	5	98.00%
Digital LSM [36]	5	99.79%
	16	92.30%
**TSWB-fMRI-SNN**	**5**	**99.30 ± 0.24%**
	**8**	**97.68 ± 0.35%**
	**16**	**96.84 ± 0.28%**

**Table 11:** Threshold-sensitivity analysis of the proposed TSWB-fMRI-SNN under different functional connectivity thresholds.

Threshold ($X_{th}$)	Network Density	Mean ± SD
0.4	**0.26 ± 0.03**	**94.62 ± 0.34%**
0.5	**0.13 ± 0.02**	**95.09 ± 0.28%**
0.6	**0.06 ± 0.01**	**94.18 ± 0.41%**

## Data Availability

The task-state fMRI data used for whole-brain functional topology construction and auditory-core circuit analysis were obtained from the English subset of OpenNeuro ds003643, Le Petit Prince: A multilingual fMRI corpus using ecological stimuli, which included 49 participants after quality control. The speech recognition experiments used the TI46 corpus (LDC93S9). Complete code reproducing the simulation results is publicly available at https://github.com/syhtsr/fMRI-SNN.git (accessed on 5 July 2026).

## Abbreviations

The following abbreviations are used in this manuscript:

fMRI	functional magnetic resonance imaging
TSWB-fMRI-SNN	Task-State Whole-Brain fMRI-constrained Spiking Neural Network
BOLD	blood oxygenation level-dependent
SNN	spiking neural network
BSA	Ben’s Spiker Algorithm
FIR	finite impulse response
DTW	dynamic time warping
FBN	functional brain network
LSM	liquid state machine
MFCC	Mel-frequency cepstral coefficient
ReSuMe	remote supervised method
STDP	spike-timing-dependent plasticity
CNN	convolutional neural network
